# Microbiota in intestinal digesta of Atlantic salmon (*Salmo salar*), observed from late freshwater stage until one year in seawater, and effects of functional ingredients: a case study from a commercial sized research site in the Arctic region

**DOI:** 10.1186/s42523-021-00075-7

**Published:** 2021-01-28

**Authors:** Jie Wang, Alexander Jaramillo-Torres, Yanxian Li, Trond M. Kortner, Karina Gajardo, Øyvind Jakobsen Brevik, Jan Vidar Jakobsen, Åshild Krogdahl

**Affiliations:** 1 Department of Paraclinical Sciences, Faculty of Veterinary Medicine, Norwegian University of Life Sciences (NMBU), P.O. Box 5003, 1432 Ås, Norway; 2 Cermaq Group AS, Dronning Eufemias gate 16, 0191 Oslo, Norway; 3 Cargill Aqua Nutrition, Prof. Olav Hanssensvei 7A, 4021 Stavanger, Norway

**Keywords:** Atlantic salmon, Commercial scale, Arctic region, Digesta-associated gut microbiota, Functional ingredients

## Abstract

**Background:**

The importance of the gut microbiota for health and wellbeing is well established for humans and some land animals. The gut microbiota is supposedly as important for fish, but existing knowledge has many gaps, in particular for fish in the Arctic areas. This study addressed the dynamics of Atlantic salmon digesta-associated gut microbiota assemblage and its associations with host responses from freshwater to seawater life stages under large-scale, commercial conditions in the Arctic region of Norway, and explored the effects of functional ingredients. The microbiota was characterized by 16S rRNA gene sequencing in distal intestinal digesta at four time points: 2 weeks before seawater transfer (in May, FW); 4 weeks after seawater transfer (in June, SW1); in November (SW2), and in April (SW3) the following year. Two series of diets were fed, varying throughout the observation time in nutrient composition according to the requirements of fish, one without (Ref diet), and the other with functional ingredients (Test diet). The functional ingredients, i.e. nucleotides, yeast cell walls, one prebiotic and essential fatty acids, were supplemented as single or mixtures based on the strategies from the feed company.

**Results:**

Overall, the fish showed higher microbial richness and lactic acid bacteria (LAB) abundance after seawater transfer, while Simpson’s diversity decreased throughout the observation period. At SW1, the gut microbiota was slightly different from those at FW, and was dominated by the genera *Lactobacillus* and *Photobacterium*. As the fish progressed towards SW2 and SW3, the genera *Lactobacillus* and *Mycoplasma* became more prominent, with a corresponding decline in genus *Photobacterium*. The overall bacterial profiles at these time points showed a clear distinction from those at FW. A significant effect of functional ingredients (a mixture of nucleotides, yeast cell walls and essential fatty acids) was observed at SW2, where Test-fed fish showed lower microbial richness, Shannon’s diversity, and LAB abundance. The multivariate association analysis identified differentially abundant taxa, especially *Megasphaera*, to be significantly associated with gut immune and barrier gene expressions, and plasma nutrients.

**Conclusions:**

The gut microbiota profile varied during the observation period, and the *Mycoplasma* became the dominating bacteria with time. *Megasphaera* abundance was associated with gut health and plasma nutrient biomarkers. Functional ingredients modulated the gut microbiota profile during an important ongrowing stage.

**Supplementary Information:**

The online version contains supplementary material available at 10.1186/s42523-021-00075-7.

## Background

The understanding of gut microbiota as a key element for proper function, health and general wellbeing of animals, including fish, has been greatly strengthened in the past decade. Although present knowledge of gut microbiota in fish is still limited [1, 2], it is clear that alterations in gut microbiota profiles may affect enzyme production [3], nutrient digestion and utilization [4, 5] and not at least the immune status which, in turn, may alter disease resistance, for better or worse (reviewed by [6–8]). Based on existing literature, it is apparent that the outcome of alterations in gut microbiota depends on complicated interactions between the host and diet composition, and not at least environmental conditions.

Most studies of gut microbiota in Atlantic salmon (*Salmo salar*) to date have described how the bacterial composition of the intestine may be affected by factors such as diet [9–13], environment (e.g. water temperature and salinity) [10, 14–18], disease situation [19], location within the digestive tract [9, 12, 20] and developmental stage [21]. For Atlantic salmon, the freshwater-to-seawater transition phase is a critical period during salmon production. The adaption to the seawater, involves a great number of physiological changes, such as increased hypoosmotic-regulatory ability, and alterations in endocrinology, metabolism, morphology and behavior [22–25]. Recent studies have also demonstrated that the freshwater-to-seawater transition can have major impacts on the microbiota communities of the intestine and skin [12, 26–28].

In Norway, Atlantic salmon production in the northernmost county, i.e. Finnmark, contributed 8.9% of the total aquaculture production in 2019 (*https://www.fiskeridir.no*). These Arctic regions are potential candidates to expand the Norwegian salmon production sites. However, present knowledge on how extreme variation in photoperiod, low average temperature, long winter period and specific pathogens in these Arctic regions influence gut microbiota is limited. Due to environmental effects on fish biology, the effect of diet composition on gut health and microbiota may differ in fish produced in Finnmark from that of fish grown in southerly areas in Norway or other countries, as stated by previous reviews [29–31]. The lack of information on how fish in Arctic conditions might differ in biology and interaction with the environment, has stimulated the feed producers to recommend use functional ingredients to strengthen the fish’ capacity to manage harsh environmental conditions and resist site-specific pathogens.

Functional ingredients, such as nucleotides, and the so-called immunostimulants and prebiotics, are commonly used to improve fish health and disease resistance, in particular during stressful farming conditions [32, 33]. They seem to have positive effects on gut health, at least under certain conditions [32, 34–36], and their effects are suggested to be mediated primarily via modulation of the gut microbiota, such as increasing numbers of beneficial bacteria (e.g. lactic acid bacteria, LAB) [6]. However, the effects of functional fish feed ingredients depend on several factors, including the characteristics of the functional ingredient itself, timing and duration of administration, fish species and life stage [6, 37, 38]. It is also likely that functional ingredients may exert different actions under practical farming conditions than what can be expected based on information from controlled tank experiments, since environmental factors possibly have greater effects than diet on fish health [39]. Yet, how functional ingredients may exert modulatory actions on gut microbiota under large scale, commercial production conditions in the Arctic areas remains relatively unexplored.

Characterizing compositional changes in intestinal bacterial communities during the production cycle, as well as exploring their associations with host responses are fundamental steps to understand the impact of gut microbiota on host functions and gut health in practical salmon production. The work presented herein is part of a larger project conducted to gain knowledge on fluctuation in gut function and health of Atlantic salmon from the late freshwater stage until 1 year in seawater farmed under large scale, commercial conditions in the Arctic areas. The host response data have been published previously [40]. The present study was conducted with three potential aims, firstly to gain knowledge on the changes in the digesta-associated gut microbiota of Atlantic salmon from the late freshwater stage until 1 year in seawater in a large-scale, commercially relevant setting under Arctic conditions in the northernmost region of Norway, secondly whether the use of functional ingredients would modify the microbiota profiles during the observation period, and finally exploring potential relationships between digesta-associated gut microbiota and host responses.

## Results

The absolute bacterial DNA level in the digesta of the distal intestine was not significantly affected by sampling time point or diet composition (*P* > 0.05, Additional file 1: Figure S1). From the 16S rRNA gene sequencing, a total number of 10.8 million counts were obtained. The minimum and maximum counts per sample were 16,639 and 159,531, respectively, with an average of 74,972 counts/sample. After sequence quality filtering, trimming and filtering of ASVs, the effective sequences were about 16,000/sample available for further downstream analyses.

### Alpha diversity

**Fig. 1:**
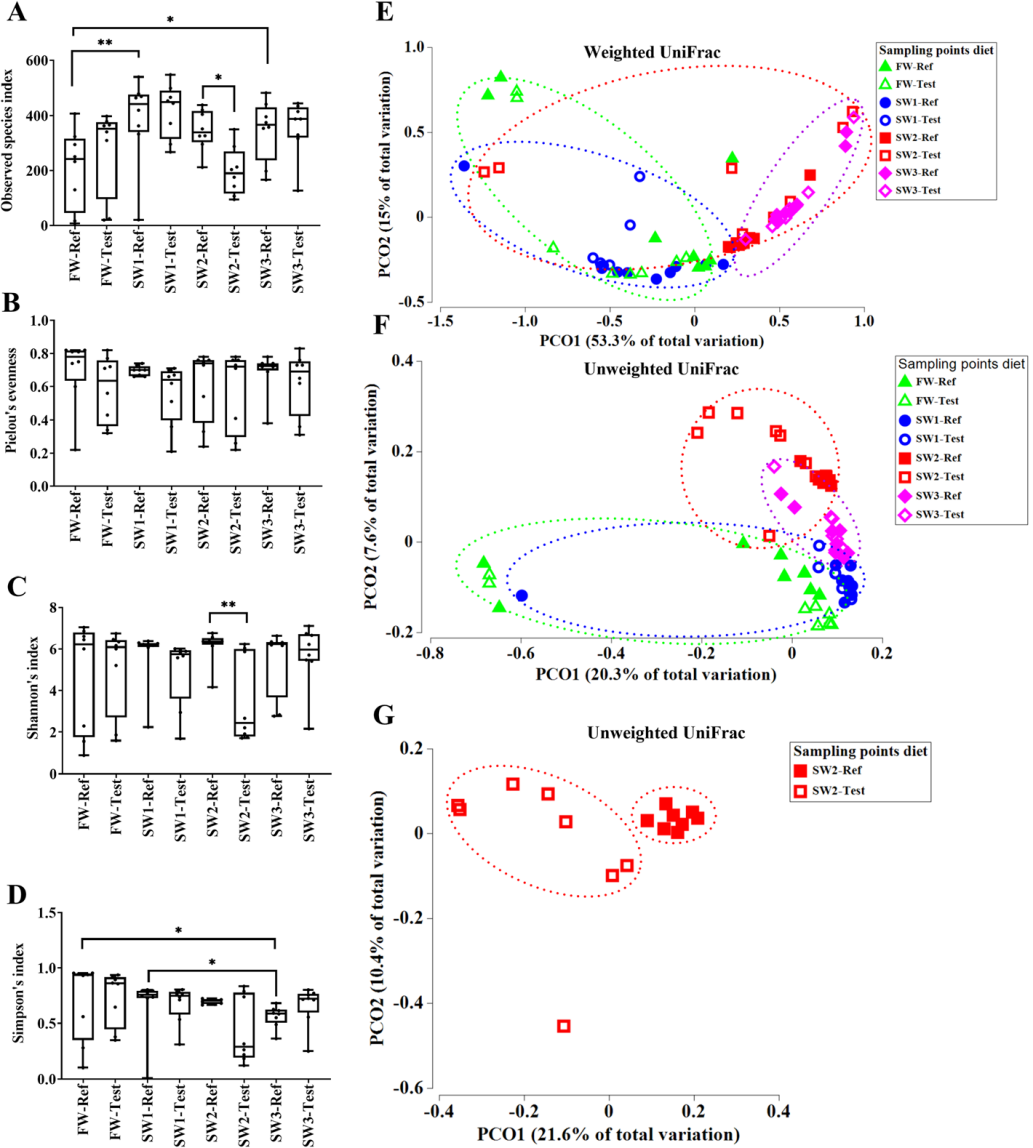
Alpha diversity and beta diversity of microbiota in digesta from the distal intestine of Atlantic salmon. **a** Microbial richness in microbiota in digesta from the distal intestine of Atlantic salmon between treatments, as measured using the Observed species index. **b** Microbial evenness in microbiota in digesta from the distal intestine of Atlantic salmon between treatments, as measured using the Pielou’s evenness. **c** Microbial diversity in microbiota in digesta from the distal intestine of Atlantic salmon between treatments, as measured using the Shannon’s index. **d** Microbial diversity in microbiota in digesta from the distal intestine of Atlantic salmon between treatments, as measured using the Simpson’s index. **e** PCoA plots based on weighted UniFrac show the clustering between treatments. **f** PCoA plots based unweighted UniFrac show the clustering between treatments. **g** PCoA plots based on unweighted UniFrac show the clustering of dietary treatment at SW2. For alpha diversity, asterisks indicate significant effect of diet among treatments (* *P* < 0.05, ** *P* < 0.01, *n* = 8). For beta diversity, each dot represents one sample

Compared to fish at FW, fish sampled from seawater showed higher microbial richness (Observed species index), especially at SW1 and SW3 (*P* < 0.05, Fig. 1a). The microbial evenness (Pielou’s evenness) (Fig. 1b) and diversity (Shannon’s index) (Fig. 1c), on the other hand, did not show significant differences between the sampling time points. However, the microbial diversity, estimated by Simpson’s index, showed a decreasing trend throughout the observation period with the lowest value in fish from SW3 (*P* < 0.05, Fig. 1d).

Regarding the effects of functional ingredients, significant differences were observed (Observed species index and Shannon’s index) at fish at SW2, but not at any of the other time points. Fish fed the Test diet showed reduced microbial richness and diversity compared to those fed the Ref diet (*P* < 0.05, Fig. 1a and c).

### Beta diversity

Results from the permutation multivariate analysis of variance (PERMANOVA) analysis of both weighted and unweighted UniFrac revealed significant differences in gut microbiota among sampling time points (*P* < 0.001, Table 1). Compared to fish at SW1, fish sampled at SW2 and SW3, showed a more apparent difference from those in fish at FW based on both weighted and unweighted UniFrac (*P* < 0.001, Table 1). Regardless of dietary treatment, principal coordinate analysis (PCoA) plots based on weighed UniFrac showed that samples at SW2 and SW3 tended to cluster together and seemed to be separated from those at FW and SW1 (Fig. 1e). The PCoA plots based on unweighted UniFrac showed that samples within each sampling time point clustered together and tended to separate from samples from other time points (Fig. 1f).

**Table 1:** Result of the PERMANOVA analysis of the weighted and unweighted UniFrac^a^

PERMANOVA	Weighted UniFrac		Unweighted UniFrac	
	Pseudo-F	*P*	Pseudo-F	*P*
Sampling time points	7.518	0.001	3.008	0.001
Pairwise comparison				
FW-Ref vs SW1-Ref		0.005		0.025
FW-Ref vs SW2-Ref		0.001		0.001
FW-Ref vs SW3-Ref		0.001		0.001
SW1-Ref vs SW2-Ref		0.001		0.001
SW1-Ref vs SW3-Ref		0.001		0.002
SW2-Ref vs SW3-Ref		0.001		0.001
Dietary effect at each sampling time point				
FW-Ref vs FW-Test	0.949	0.409	1.056	0.347
SW1-Ref vs SW1-Test	2.331	0.05	1.271	0.01
SW2-Ref vs SW2-Test	2.099	0.129	2.937	0.001
SW3-Ref vs SW3-Test	0.698	0.406	1.176	0.146

^a^FW, sampling time point in freshwater (May 2016); SW1, the first seawater sampling time point (June 2016); SW2 the second seawater sampling time point (November 2016); SW3, the final seawater sampling time point (April 2017); Ref: diet without functional ingredients; Test, diet with functional ingredients

Significant effect of dietary treatment, i.e. inclusion of functional ingredients, was observed in fish at SW1 and SW2 according to the PERMANOVA analysis of unweighted UniFrac (*P* < 0.01 and *P* < 0.001, respectively, Table 1). The PCoA plots based on unweighted UniFrac metrics showed that at SW2 fish within the Ref diet tended to cluster together compared to those in Test-fed fish (Fig. 1g). No clear diet effects were observed in fish at FW or SW3 (*P* > 0.05, Table 1). Overall, fish from the SW2 sampling time point showed the strongest response to diet, at which significant effects on both alpha and beta diversity were observed. We, therefore, have chosen to present details regarding the effects of functional ingredients on microbiota assemblage for SW2 only.

### Digesta-associated gut microbiota composition

**Fig. 2:**
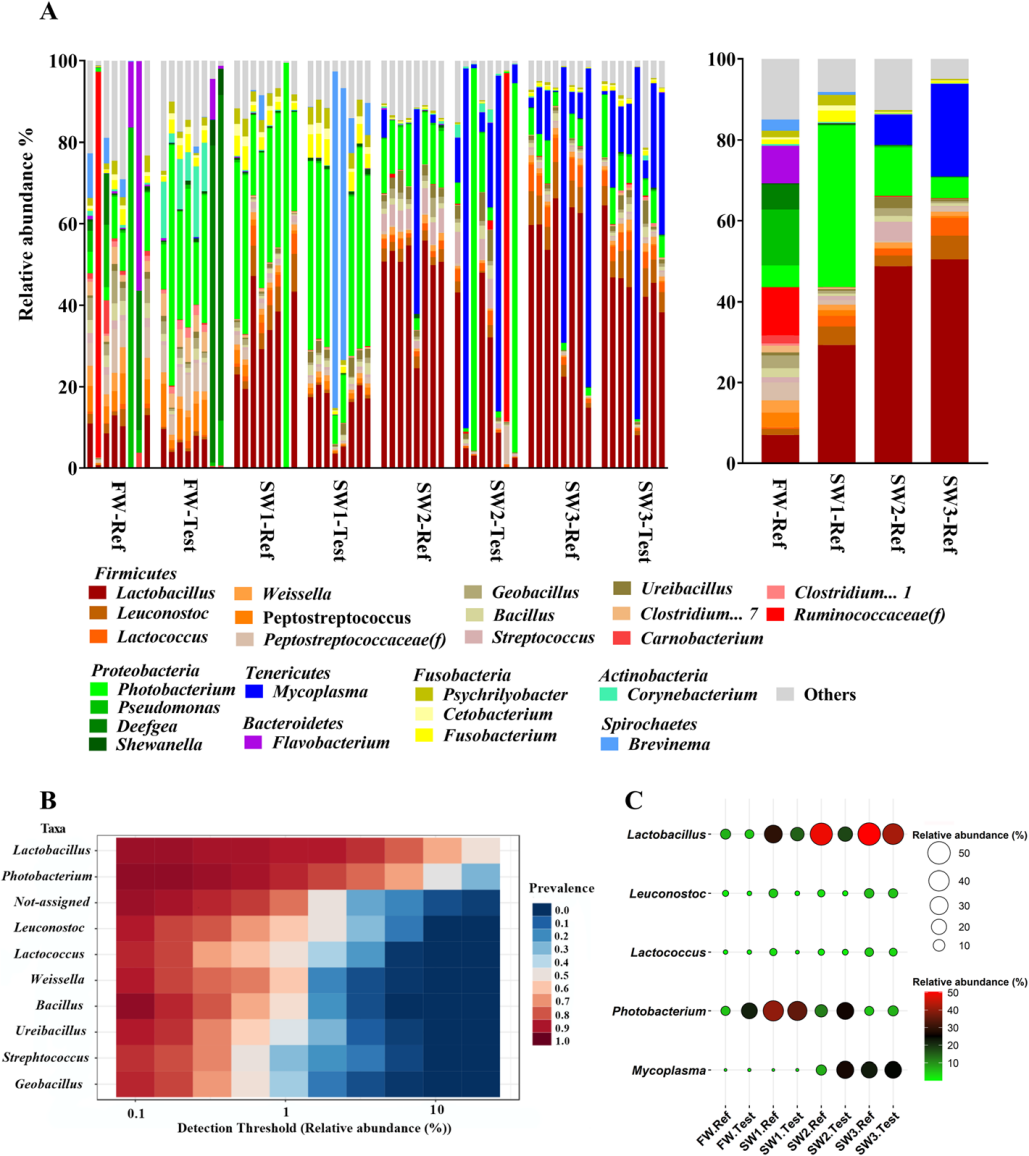
Digesta-associated distal intestinal microbiota composition of Atlantic salmon. **a** Top 25 most abundant taxa at genus level of all samples and mean (right side) relative abundance of each taxon between fish fed Ref diet among sampling time points. The top 25 genera were selected accounted for more than 80% of the total abundance in each treatment. f__, family. **b** The core microbiota between samples at genus level. The figures showing the bacteria were selected above 0.1% relative abundance in 80% of samples. **c** Balloon plot showing the relative abundance of five major genera between treatments (*n* = 8). The five major genera were selected based on MaAsLin 2 and core microbiota analysis

Twenty-seven different phyla were identified for all samples (Presented in Additional file 2: Table S1). The relative abundance of microbiota in all samples at the genus level (the top 25 genera) is illustrated in Fig. 2a. Overall, the most abundant phyla *Firmicutes* and *Proteobacteria* varied among sampling time points, and accounted, in total, for about 80% of the total abundance. At FW and SW1, the composition of digesta-associated gut microbiota was strongly dominated by the phylum *Firmicutes* and *Proteobacteria*. The *Firmicutes*/*Proteobacteria* ratio was 1.8 and 1.1 at FW and SW1, respectively. As fish progressed towards SW2 and SW3, phyla *Firmicutes* and *Tenericutes* became more prominent corresponding to a decline in phylum *Proteobacteria*. The *Firmicutes*/*Proteobacteria* ratio increased value between SW2 to SW3, from 4.2 to 10.1, while the *Firmicutes*/*Tenericutes* ratio decreased from 10 to 3 during the same time interval.

**Fig. 3:**
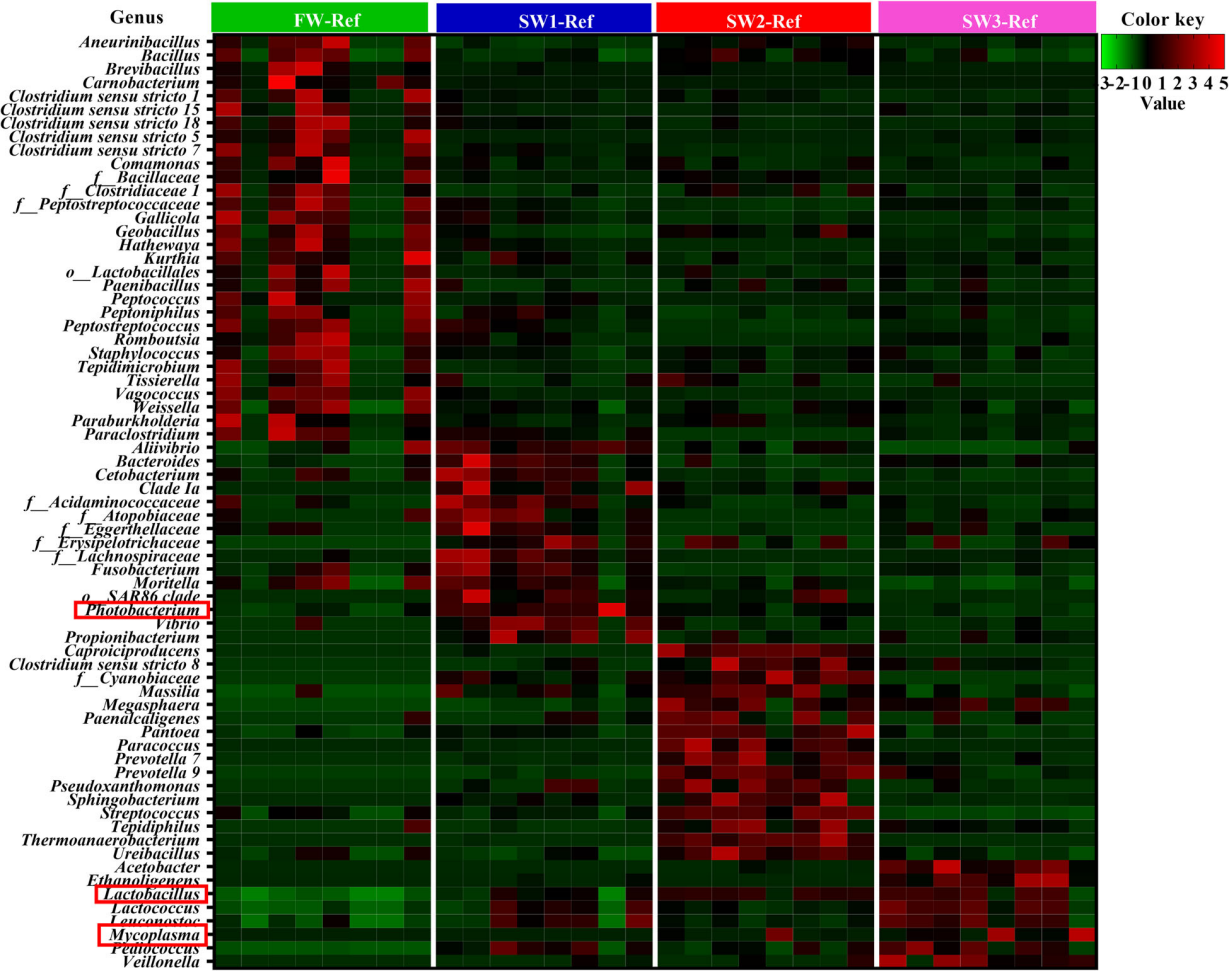
Heatmap of bacterial abundance in fish fed the Ref diets based on MaAsLin 2 analysis. Rows indicate results for 69 bacteria at genus level (*q-value* < 0.05), columns depict the results for the 8 samples at each of the four sampling time points. Color differences indicate differences in normalized relative abundances at genus level, i.e., red (high positive value) indicates the maximum relative abundance; Green (low negative value) indicates the minimum relative abundance. o__: order; f__: family

The most abundant genera within the phylum *Firmicutes* were lactic acid bacteria (LAB), mainly *Lactobacillus*, *Leuconostoc* and *Lactococcus*, and the most abundant genera within *Proteobacteria* and *Tenericutes* were *Photobacterium* and *Mycoplasma*, respectively (Fig. 2a). Across all samples, 10 genera including *Lactobacillus, Photobacterium, Leuconostoc* and *Lactococcus*, were core microbiota at genus level (above 0.1% relative abundance in 80% of samples) (Fig. 2b). Notably, at FW, genera *Deefgea*, *Flavobacterium* and *Pseudomonas*, as well as family *Ruminococcaceae* were only detected in a few fish, but when present, they dominated the gut microbiota (Fig. 2a). Here, we focus on the three major genera, i.e. *Lactobacillus*, *Photobacterium* and *Mycoplasma*, as they varied among sampling time points. Specifically, after seawater transfer, fish showed an increased relative abundance of *Lactobacillus*, i.e. 29% ± 15, 48% ± 10 and 50% ± 20% at SW1, SW2 and SW3, respectively, compared to that those at FW (7% ± 6%) (Fig. 2a and c). Regarding *Photobacterium*, 4 weeks after seawater transfer, i.e. at SW1, fish had the highest relative abundance of *Photobacterium* (40% ± 25%) compared to fish from other sampling time points (Fig. 2a and c). The higher relative abundance of *Mycoplasma* was observed in fish at SW2 (7% ± 17%) and SW3 (23% ± 31%), while low levels, less than 0.1%, were observed at FW and SW1 (Fig. 2a and c). The MaAsLin 2 analysis showed significant differences in 69 genera among sampling time points including *Lactobacillus*, *Photobacterium* and *Mycoplasma* (Fig. 3).

**Fig. 4:**
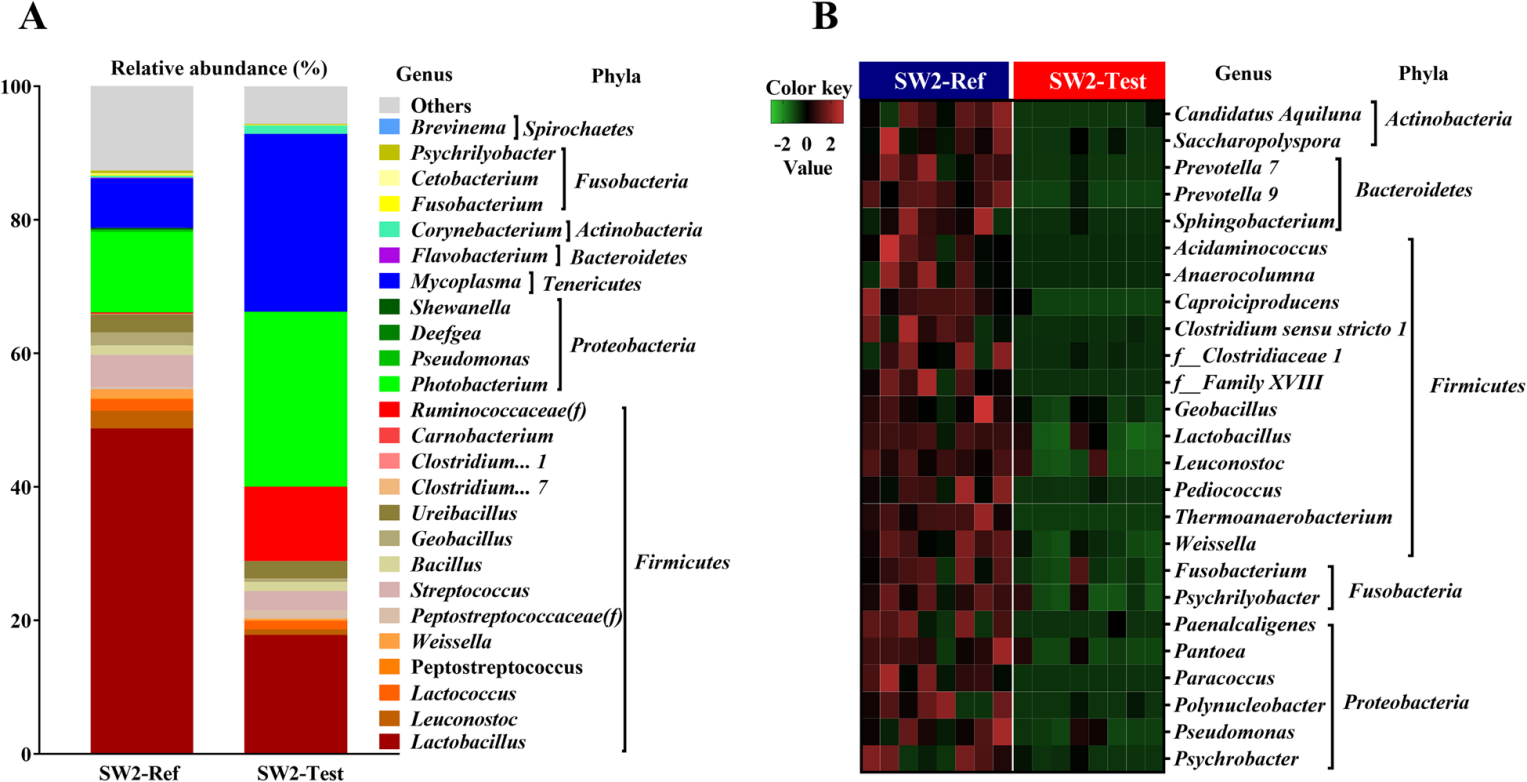
The effect of dietary functional ingredients on microbiota in digesta from the distal intestine of Atlantic salmon at SW2. **a** Mean relative abundance (Top 25) of each taxon at genus level in fish at SW2 fed Ref and Test diets. The top 25 genera were selected accounted for more than 80% of the total abundance in each treatment. **b** The heatmap of bacterial abundance at SW2 based on MaAsLin 2 analysis. Rows depict results for 25 bacteria (*q-value* < 0.05), columns depict the results from the 8 samples from fish fed each of the two diets. Colors correspond with normalized relative abundances, i.e., red (high positive value) indicates the maximum relative abundance; Green (low negative value) indicates the minimum relative abundance. f__: family

Regarding the effects of functional ingredients at SW2 (Fig. 4a), Test-fed fish had a lower relative abundance of *Lactobacillus* (17% ± 19%) than those in Ref-fed fish (48% ± 10%). The relative abundance of *Photobacterium* (12% ± 4%) and *Mycoplasma* (7% ± 17%) were observed in Ref-fed fish, while *Photobacterium* (26% ± 40%) and *Mycoplasma* (27% ± 37%) were found in Test-Fed fish. The MaAsLin 2 analysis showed that significant differences between Ref and Test diets were due to the decrement of 25 genera/family in fish fed Test diet, including LAB, such as *Lactobacillus* and *Leuconostoc* (Fig. 4b).

### Significant associations between microbiota of distal intestinal digesta and host responses

Of note, the gut immune gene expression levels were negatively correlated with their PC1 value of the PCA, while gut barrier gene expression and plasma nutrient levels were both positively related with their PC1 value of the PCA (Additional file 3: Table S2).

**Fig. 5:**
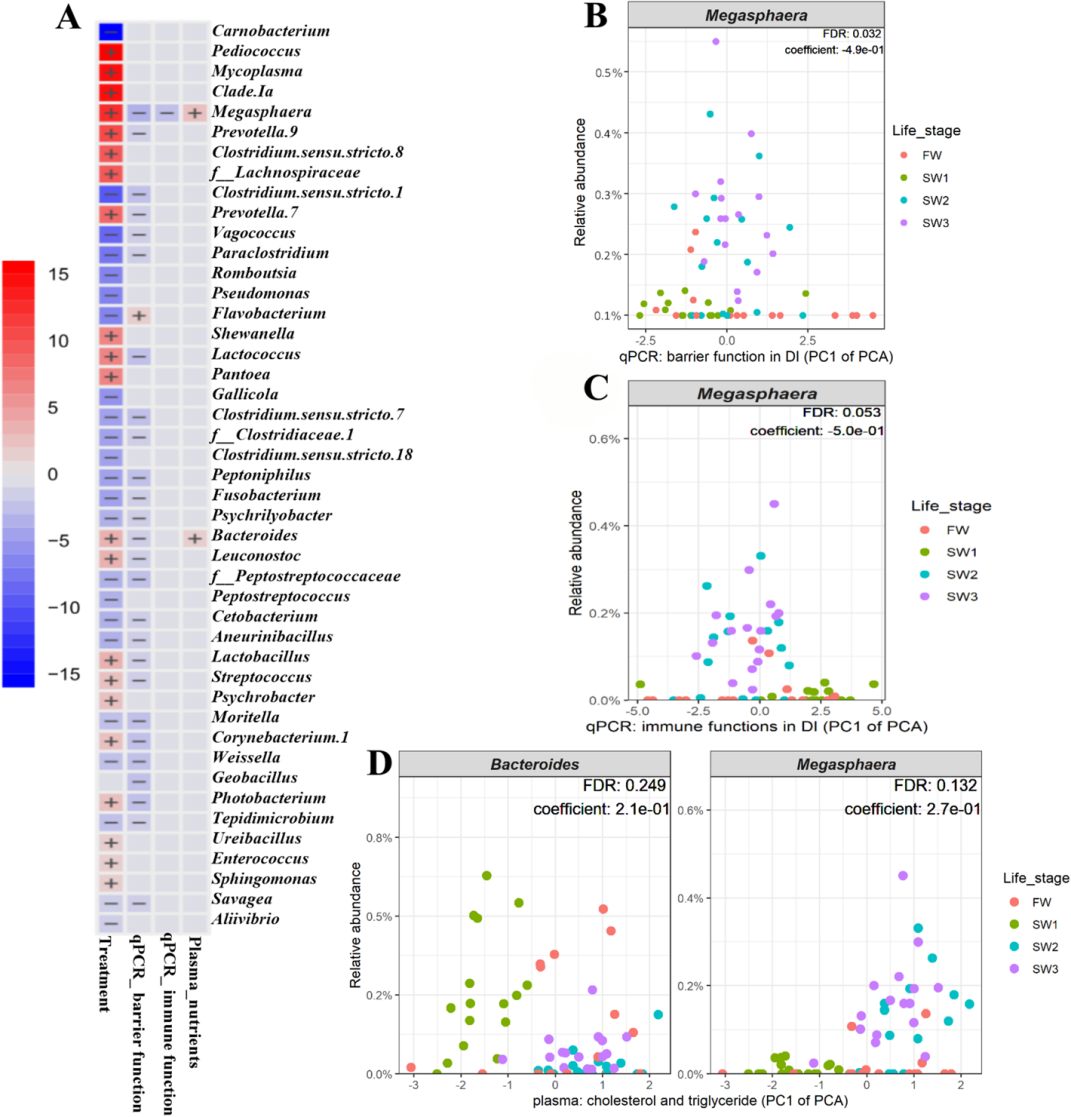
Significant associations between microbial clades with sample metadata. **a** Heatmap summarizing all the significant associations between microbial clades and sample metadata. Color key: -log (q-value) * sign (coefficient). Cells denoting significant associations are colored (red or blue) and overlaid with a plus (+) or minus (−) sign indicating the direction of association: qPCR_barrier_function (−), negative correlation between microbial clade abundance and qPCR_barrier_function (PC1 of PCA); qPCR_immune_response (−), negative correlation between microbial clade abundance and qPCR_immune_function (PC1 of PCA); plasma_nutrients (+), positive correlation between microbial clade abundance and the levels of plasma_nutrients (PC1 of PCA). **b** The negative correlation between the relative abundance of *Megasphaera* and qPCR_barrier_function (PC1 of PCA). **c** The negative correlation between the relative abundance of *Megasphaera* and qPCR_barrier_function (PC1 of PCA). Of note, the gut immune gene expression levels were negatively correlated with PC1 of the PCA, which decreased as PC1 of the PCA increased (Additional file 3: Table S2). Hence, the relative abundance of *Megasphaera* shows a positive correlation with immune gene expression level. **d** The positive correlation between the relative abundance of *Megasphaera* and *Bacteoides* and plasma_nutrients (PC1 of PCA), respectively. FDR, false discovery rate; f__, family. The significant association was set at FDR < 0.25

The multivariate association analysis identified 27 differentially abundant taxa that were significantly correlated with gene expression related to gut barrier function (Fig. 5a). Except for *Flavobacterium*, 26 differentially abundant taxa, such as *Megasphaera*, *Photobacterium* and LAB (e.g. *Lactobacillus*), showed a negative correlation with expression levels related to barrier function genes (diagnostic plots of raw data in Additional file 1: Figure S2). For example, the relative abundance of *Megasphaera* showed a clear negative correlation with expression levels of DI barrier function genes, which decreased as PC1 of the PCA increased (FDR = 0.032, coefficient = − 0. 49, Fig. 5b).

The relative abundance of *Megasphaera* was negatively correlated with PC1 of the PCA of immune functions (FDR = 0.053, coefficient = − 0. 5, Fig. 5a and c), which decreased as PC1 of the PCA increased. Hence, the relative abundance of *Megasphaera* was positively correlated with gut immune gene expression levels.

The relative abundance of *Megasphaera* (FDR = 0.132, coefficient = 0. 27) and *Bacteroides* (FDR = 0.249, coefficient = 0.21) showed weak positive correlations with the levels of plasma nutrients, which increased as PC1 of the PCA increased (Fig. 5a and d).

## Discussion

### Effects of sampling time points on microbiota in digesta from the distal intestine

The observed changes in gut microbiota composition from freshwater to seawater sampling points were probably related to the adjustments made in commercial diets, different environmental conditions across sampling points, as well as the host physiology, which changed substantially during the observation time, may play important roles in these alterations. Together, these changes likely lead to the competitive distribution by certain microbial species, thereby reorienting the digesta-associated gut microbiota composition of the fish. Regarding environmental conditions, the alterations in water temperature and salinity are well documented to influence gut microbiota [14, 30, 41]. Also, the freshwater-to-seawater transition has profound effects on host physiology, e.g. on osmoregulatory and immune functions in the gut [22, 42–44]. The effects of physiological changes on gut microbiota profiles in our study may be a protracted process taking longer than the four-week timeline examined, and are generally in line with recent studies in Atlantic salmon [12, 28].

Although previous studies have reported that the freshwater-to-seawater transition has a major effect on the microbiota profiles of gut digesta in salmon, the observed changes in intestinal microbiota composition vary inconsistently among studies [12, 26, 27, 45]. Our observation that fish showed an increase in microbial richness in distal intestinal digesta after seawater transfer is in agreement with the results of previous studies of the gut [27] and skin microbiota [28] of Atlantic salmon. However, other studies have reported that seawater transfer decreases [26, 45] or maintains [12, 21] microbial richness compared to the freshwater stage. In the present work, the phyla *Firmicutes* (mainly genus *Lactobacillus*) and *Proteobacteria* (mainly genus *Photobacterium*) dominated the gut microbiota 4 weeks after seawater transfer, which is in accordance with the study of Lokesh and co-workers [21]. Other studies have reported that the phylum *Firmicutes* strongly dominate the microbiota of distal intestinal digesta, whereas other taxa, including phylum *Proteobacteria*, decline 3 weeks after seawater transfer [26, 27]. The reasons behind this discrepancy are unclear but are likely related to variation in environmental conditions, diet composition and sample origin, and possibly also methodology.

The LAB has been identified as major component of the gut microbiota in Atlantic salmon and is presumed to have beneficial effects on the host through immune regulation, improvement of digestive processes and inhibition of pathogens, at least under some conditions [46–49]. Our study showing a higher relative abundance of LAB (mainly *Lactobacillus*) in fish during seawater stages than those in FW, is in agreement with the results of Dehler and co-workers [26]. In the present study, the alteration in LAB abundance occurred in parallel to the increase in content of plant ingredients in the diets at SW2 and SW3. The increased dietary content of fermentable plant carbohydrates, serving as a substrate for LAB bacteria, may therefore be the cause of the increase in the relative abundance of LAB, as also observed in previous studies [9, 20, 50, 51]. However, one recent study found a significant decrease in the relative abundance of LAB in mucosa-associated microbiota of Atlantic salmon 6 weeks after seawater transfer [12]. Digesta- and mucosa-associated gut microbiota have been shown to vary substantially in composition [9, 12, 20]. It is therefore possible that these two gut compartments may contain different LAB levels and/or compositions. Given the potential functional role of LAB for salmon health, additional studies of both digesta- and mucosa-associated microbiota are recommended to increase our knowledge of LAB roles on salmon health and function.

Compared to fish at SW2 and SW3, the bacterial taxa of fish at SW1 were more similar to the taxa at FW. This may be related to the fact that it takes time to colonize the intestine [52]. There was only 7 weeks between the FW sampling time point and SW1, whereas the SW1 and SW2 samplings were conducted after 18 weeks intervals. The alteration in microbial profile from SW1 to SW2 may have other potential causes. Since the observation period (from June to November) between SW1 and SW2 is an important stage for growth and physiological changes due to increased water temperature and high feed intake [40], the temperature may be the leading environmental factor impacting these alternations, and diet could exert synergistic effects. Day length is another environmental factor that might have influenced gut microbiota in our study, for which documentation is lacking so far.

As the Atlantic salmon progressed towards the adult stage, the enrichment of genus *Mycoplasma* at SW2 and SW3 time points compared to FW and SW1 was one of the most prominent differences found in our study, possibly indicating that the host is a determinant for microbial assemblage by attracting specific bacterial communities depending on the developmental stage of the fish [53, 54]. Previous studies have found *Mycoplasma*, a member of the core microbiota, to be one of the most abundant bacteria in farmed as well as wild Atlantic salmon during seawater stages [26, 55–59] reaching levels above 70% of total abundance in certain cases [19, 45, 58]. Increased levels of genus *Mycoplasma* with time may therefore be an important characteristic for gut microbiota of adult Atlantic salmon. As *Mycoplasma* is rarely [52] or not [19, 59] observed in samples from surrounding seawater, their enrichment may be independent of rearing habitat. The reason for their colonization in the intestine is uncertain although they seem to be particularly well-adapted to the intestinal environment of Atlantic salmon. Recently, potential functional roles of *Mycoplasma* in Atlantic salmon have been suggested. For example, *Mycoplasma* abundance was strongly associated with flesh color darkness, suggesting a role in the production of carotenoids [60]. Moreover, the *Mycoplasma* has shown ecological and functional associations with the host [61] and is positively correlated with body weight [54]. However, the present study did not show a significant association between the relative abundance of *Mycoplasma* and the observed host responses. Given the indicated important relationship between *Mycoplasma* and host [61], more studies are warranted to increase knowledge of their functional significance, including potential probiotic applications to Atlantic salmon.

### Effects of functional ingredients on microbiota in digesta from distal intestine

Available research indicates that functional ingredients, when included in fish diets, such as prebiotics, nucleotides and immunostimulants, may affect gut microbiota through direct or indirect modulatory effects (reviewed by [6, 37–39, 62, 63]). However, except at SW2 in our study, the applied functional ingredients were unable to produce significant alterations in the microbial profile of the distal intestine. How dietary functional ingredients may modulate gut microbiota composition in fish will depend on various important factors, such as the specific composition of the functional ingredients, timing and duration of administration, fish life stage, fish physiology, as well as environment factors [37, 38]. The lack of effects of functional ingredients could be explained by low feed intake, since our study was conducted under the harsh Arctic conditions with the low average temperature during most of the observation period. Another explanation is that compared to the small-scale experimental trials of limited duration in the majority of available scientific literature, it is likely that, the complicated and changeable environmental conditions in the current study may have resulted in the diminished impact of the functional ingredients on gut microbiota [39].

As mentioned above, the observation period from SW1 to SW2 is an important ongrowing stage due to high temperature and long daylight, and thereby high feed intake. The decreased microbial richness and diversity and the relative abundance of LAB, observed at SW2 for Test-fed fish could therefore be attributed to the high ingestion of a mixture of nucleotides, yeast cell walls and essential fatty acids. Similar results have been observed in rainbow trout previously [64]. Unexpectedly, these findings seem to be in contrast to the positive effects often ascribed to functional ingredients on the abundance of presumed beneficial bacteria, such as LAB, in the intestine and other mucosal surfaces [6, 38, 65–67]. One potential explanation for the discrepancy may be linked to the duration of administration as long-term oral administration of immunostimulants have been reported to cause decreased efficacy in fish [68]. This assumption is also supported by an apparent increased metabolic cost in fish fed these functional ingredients at SW2, i.e. lower condition factor and plasma triglyceride levels, and a tendency to lower growth [40]. It is, therefore, possible that the ten-week continuous oral administration of these selected functional ingredients before the sampling at SW2, may have resulted in less favorable changes in gut digesta microbiota composition, and may explain the reduced richness, diversity and relative LAB abundance. Since there are indications that the increased population of LAB may not always be beneficial for salmon [9, 20, 50, 51], it is too early to conclude that Ref-fed fish showed a “healthier” gut microbiota profile compared to Test-fed fish. The interaction between gut microbiota and the host is too complex to generalize regarding which microbiota profile may benefit or be detrimental to the host physiology [3]. One example can illustrate this; certain LAB, such as the probiotic strain *Lactobacillus plantarum*, may induce gut dysbiosis in fish [69] and disrupt healthy intestinal tissues in mammals [70, 71]. Our observations showing that the LAB abundance was negatively correlated with gut barrier gene expression suggest weakened barrier functions in fish with a high population of LAB. Altogether, observations gathered so far points to a need for more information on how to use functional ingredients for optimal fish health and performance.

### Associations between microbiota of distal intestinal digesta and host response

The gut microbiota can be divided into digesta- and mucosa-associated microbiota based on their location in the intestine. Compared to the digesta-associated gut microbiota, the mucosa-associated gut microbiota refers to microorganisms closely associated with intestinal epithelial cells, and thereby may play a more vital role in gut mucosal immunology, energy homeostasis and nutrient metabolism [29, 72]. However, the lack of attachment of the digesta-associated gut microbiota to the mucosa does not mean marginal importance for the host [73]. The digesta-associated microbiota has been linked to modulation of host functions through their metabolite production, such as the production of short-chain fatty acids and polysaccharides [74, 75]. We found 26 digesta-associated taxa including *Photobacterium*, LAB and *Megasphaera* to be negatively correlated with gut barrier gene expression, while *Megasphaera* was positively associated with gut immune gene expression and plasma nutrient levels. This is clearly indicative of relationships between digesta-associated gut microbiota and salmon gut functions, which should be explored in targeted future studies.

The genus *Photobacterium* is often reported to belong to the core bacteria of the Atlantic salmon gut [9, 11, 12, 16, 20, 21, 27]. In the current study, a negative relationship between the expression levels of barrier function genes and the relative abundance of genus *Photobacterium* suggests a weakening of the barrier function in fish with high *Photobacterium* abundance*.* In this context, it is to be noted that previous studies have demonstrated that certain *Photobacterium* species may cause disease outbreaks in salmon right after seawater transfer, and that this may be linked to malfunction of the gut barrier [76, 77]. These results call for further studies to strengthen the understanding of relationships between *Photobacterium* species, gut barrier functions, and disease resistance in salmon.

Our findings also demonstrated that the relative abundance of *Megasphaera,* a genus of the phylum *Firmicutes*, was positively correlated with expression levels of gut immune genes, and negatively with expression levels of barrier related genes. Published studies revealing the effects of gut microbiota on immune and barrier responses in Atlantic salmon are still limited. The mechanism behind and implications of these relationships remain unknown. Possibly, certain bacterial taxa shape intestinal barrier and immune functions, and could thereby regulate metabolic functions [78]. The credibility of such associations will, in theory, increase with increasing sample size, and future studies with larger sample size are therefore warranted.

## Conclusions

This study provides new information on the dynamics of salmon digesta-associated gut microbiota assemblage and its associations with host responses from the late freshwater stage until 1 year in seawater during large-scale, commercial farming conditions in the northernmost region of Norway. The core microbiota, genera *Lactobacillus* and *Photobacterium*, varied among sampling time points. As fish progressed towards adult, the genera *Lactobacillus* and *Mycoplasma* became more prominent corresponding to a decline in genus *Photobacterium* indicating more apparent separation with fish from freshwater. Significant effect of functional ingredients on gut microbiota was observed at fish after a rapid growth period showing that inclusion of a mixture of nucleotides, yeast cell walls and essential fatty acids reduced microbial richness and diversity, as well as the relative abundance of LAB. The differentially abundant taxa including *Photobacterium,* LAB (e.g. *Lactobacillus*) and *Megasphaera* were found to be negatively correlated with gut barrier gene expression, while the relative abundance of *Megasphaera* was positively correlated with the levels both in gut immune gene expression and plasma nutrients.

## Materials and methods

### Experimental fish

**Fig. 6:**
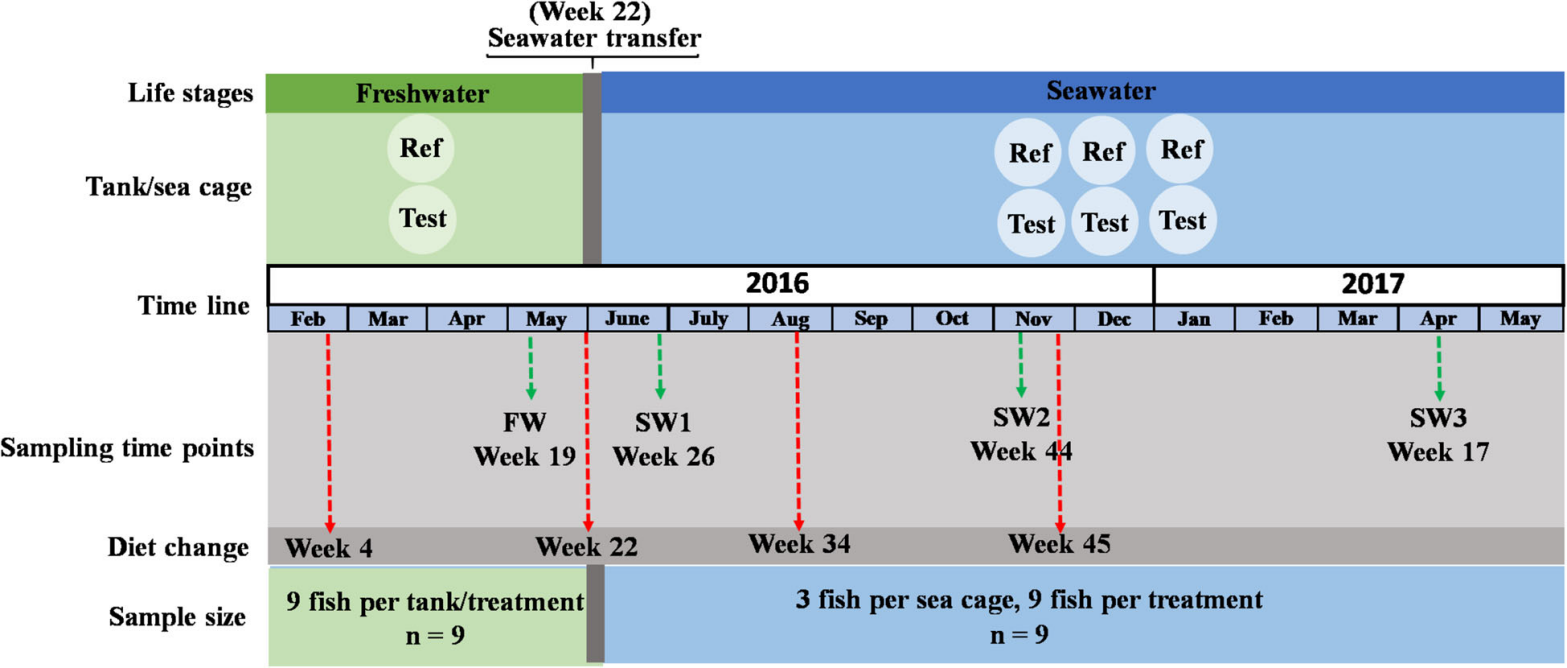
Outline of the sampling program. The four diet series were changed at week 4, week 22, week 34 and week 45, respectively (Diet composition see Table 2)

The experimental setup is shown in Fig. 6. Atlantic salmon hatched in the spring of 2015 were reared in two large, closed aluminum flow-through tanks for spring smolt production at Hopen, near Bodø of Norway (N67° – E14°). The tanks were supplied with freshwater from a nearby lake. When the fish were ready to be transferred to seawater, they were transported by a well-boat to Sommarbukt (N70° – E22°), in Finnmark county of Norway, where the fish from each tank were split into triplicated sea cages, i.e. three replicates for each dietary treatment in seawater, each holding about 55,000 fish. The temperature followed natural fluctuations in the water intake, ranging from 1 to 14 °C for the entire period. Oxygen and salinity levels fluctuated from 8 to 15 mg/L and from 11 to 44 ‰ throughout the experimental period, respectively (Additional file 1: Figure S3).

### Diet composition and sampling

The macronutrient composition of the diet series varied throughout the observation time according to the requirements of the fish. At each observation time, two series of diets were fed, one without functional ingredients (Ref diet) and one with functional ingredients (Test diet). The functional ingredients, e.g. nucleotides, yeast cell walls, a prebiotic and essential fatty acids, were supplemented to the diets either as a single ingredient or as mixtures following the strategy developed for this particular commercial site and according to the development and production stage of the fish in the farm. The inclusion levels of these functional ingredients were not listed due to commercial interests and the production of intellectual rights. The samples were collected at four sampling time points as the experimental set-up in Fig. 6: 2 weeks before seawater transfer (May 2016, FW) and three times during the seawater period, i.e. 4 weeks after seawater transfer (June 2016, SW1) and two times thereafter (November 2016 and April 2017, SW2 and SW3, respectively). Eight treatments were defined by sampling life stages and dietary treatment, i.e. FW-Ref, FW-Test, SW1-Ref, SW1-Test, SW2-Ref, SW2-Test, SW3-Ref and SW3-Test. The formulations and nutrient compositions of the diets among treatments are presented in Table 2.

**Table 2:** Feed composition and formulation of diets during the feeding trial^a^

Feed composition	FW-Ref	FW-Test	SW1-Ref	SW1-Test	SW2-Ref	SW2-Test	SW3-Ref	SW3-Test
**Ingredients (%)**								
Marine protein sources^b^	40	40	30	30	19	19	19	19
Plant protein sources^c^	35	35	39	39	53	53	53	53
North Atlantic fish oil	9	9	24	24	10	10	10	10
Rapeseed oil	9	9	–	–	7	7	7	7
Binders & Micronutrients	7	7	7	7	11	11	11	11
Sum	100	100	100	100	100	100	100	100
**Nutrient composition (%)**								
Crude protein	44	44	44	44	46	46	46	46
Crude fat	22	22	28	28	22	22	22	22
Starch	7.5	7.5	8	8	10	10	10	10
Crude fiber	1.5	1.5	3	3	3	3	3	3
Ash	7	7	6	6	5	5	5	5
**Functional ingredients**^d^								
Essential fatty acids	–	–	–	√	–	√	–	√
Nucleotides	–	√	–	√	–	√	–	√
Yeast cell walls	–	–	–	–	–	√	–	–
A prebiotic	–	–	–	–	–	–	–	√

^a^The composition of four different basic diets varied throughout the time of observation following the strategy developed for this commercial site according to the development and production and health of the fish in the farm. At each observation time, two series of diets were fed, one without functional ingredients (Ref diet) and one with functional ingredients (Test diet). FW, sampling time point in freshwater (May 2016); SW1, the first seawater sampling time point (June 2016); SW2 the second seawater sampling time point (November 2016); SW3, the final seawater sampling time point (April 2017). ^b^Mix of Scandinavian origin fish meal and, Fish protein concentrate (Norway). ^c^Mix of soy protein concentrate, wheat protein concentrate, wheat gluten, sunflower meal. ^d^Inclusion levels were determined according to recommendations from the producers and cannot be disclosed due to commercial interests and intellectual rights

Only fish with digesta throughout the distal intestine were selected to ensure exposure to the diet at the time of sampling. At each sampling time point, 3 times 3 fish were sampled for each dietary treatment. Regarding the freshwater sampling, three groups of fish came from the same tank as the facility’s tanks were too big, each holding 180,000 fish, and the facility did not allow replicate tanks for each diet. This approach was considered to be suitable and included in the statistical evaluation as independent replicates for observation of diet effects. The results of our study confirmed that this approach was acceptable, as the means of the fish in the two tanks did not differ significantly, and the variances were similar, indicating no important tank variation [40]. For the sampling in seawater, the three groups of fish per diet came from three sea cages. A total of 72 fish were collected for DNA extraction. All tools were cleaned and decontaminated by an ethanol spray and flaming during each sampling fish. For digesta-associated gut microbiota analysis, only distal intestinal digesta (also called chyme), as previously defined [79], was collected into 1.5 mL skirted sterile centrifuge tubes, then mixed thoroughly using a spatula before frozen in liquid N_2_, thereafter stored at − 80 °C before DNA extraction.

### DNA extraction

One fish was randomly selected from per treatment to divide 72 samples into 9 batches for DNA extraction. About 100 mg of digesta of distal intestine from each sample was used for DNA extraction and processed according to the protocol in the QIAamp Fast DNA Stool Mini Kit (Qiagen, Hilden, Germany), except for an additional heating step following the bead beating step at 95 °C for 7 min before proceeding according to the standard procedure suggested by [80]. At each DNA extraction batch, a blank negative control and positive mock control (ZymoBIOMICS Mock Community Standard, Zymo Research Corp, Irvine, CA, USA) were included and processed in parallel with the experimental samples.

### PCR amplification

PCR amplification of about 300 bp amplicons from the V1-V2 region of the 16S rRNA was carried out using the bacterial universal primers 27F (5′ AGA GTTTGA TCM TGG CTC AG 3′) and 338R-I (5′ GCW GCC TCC CGT AGG AGT 3′) and 338R-II (5′ GCW GCCACC CGT AGG TGT 3′). The PCR was carried out as previously described by Gajardo et al. [20] using 25 μl sample volume in duplication with 2 μl of DNA template, 22.4 μl Phusion® High-Fidelity PCR Master Mix (Thermo Scientific, CA, United States of America) and 0.6 μl of forward (27F) and reverse (pooled 338R-I and II) primers (50 pM). The PCR was run in duplicate and negative PCR controls using molecular grade water as a template were included. The duplicate PCR products were pooled and analyzed in 1.5% agarose gels and samples with bright bands between 300 and 350 bp were considered suitable for further processing. Since samples from one of 9 batches showed the low quality of PCR products, we removed these samples for further analysis. Hence, there were 8 samples per treatment left for final sequencing (*n* = 8).

### DNA quantification

The 16S rRNA gene quantity in the diluted DNA templates used for the amplicon PCR was measured by qPCR. The qPCR assays were performed using a universal primer set (forward, 5′-CCA TGA AGT CGG AAT CGC TAG-3′; reverse, 5′-GCT TGA CGG GCG GTG T-3′) used for bacterial DNA quantification as the description in previous studies [81, 82].

### PCR cleanup, library preparation and sequencing

PCR cleanup, library preparation and sequencing were performed using the protocol provided by Illumina (part #15044223 Rev. B). Briefly, the PCR products were cleaned using AMPure beads followed by index PCR using Nextera XT Index kit (Illumina, California, USA; catalog no., FC-131-1096) and subsequently another round of purification with the AMPure beads. After the cleanup, the representative libraries were analyzed using the Agilent DNA 1000 Kit (Agilent Technologies, California, USA; catalog no., 5067–1505) to verify the library size. The cleaned libraries were quantified using a Qubit fluorometer (Thermo Scientific, CA, United States of America). The library was then denatured and diluted to 6 pM, 20% of 6 pM PhiX control was added before finally being sequenced on an Illumina MiSeq platform. 300 bp paired-end reads were generated.

### Data analysis

Raw sequence data were analyzed using the Quantitative Insights Into Microbial Ecology 2 (QIIME 2) software version 2019.4 (https://qiime2.org/) [83]. These sequence data were processed using the DADA2 algorithm in QIIME2 to generate amplicon sequence variants (ASVs) [84]. The demultiplexed paired-ended reads were analyzed using QIIME2. The reads were trimmed off the primer sequences (the first 20 bps for forward reads; the first 18 bps for reverse reads), truncated where the median Phred quality score crashed (250 bp for forward reads; 190 bp for reverse reads). Then, the low-quality reads were filtered off. The taxonomy was assigned in QIIME2 against the SILVA database (version 132) [85] trained with a scikit-learn naive Bayes machine-learning classifier [86]. The contaminant sequences were removed based on their prevalence and abundance in the samples according to previous descriptions [87]. The majority of removed sequences were classified as *Pseudomonas, Acinetobacter, Leptothrix, Aeromonas,* an unclassified bacterium of *Betaproteobacteriales* order, three kinds of genera *Flavobacterium*, an unclassified bacterium of *Chitinophagales* order and *Cutibacterium*. *Streptophyta* filtering is usually performed to remove chloroplast sequences which are assumed to reflect non-bacterial-associated taxa [88]. The other sequences considered as contamination were sequences found in the negative controls from both de DNA extraction and PCR amplification.

### Phylogenetic classification, richness and diversity parameters

All ASVs were aligned with MAFFT [89] and then phylogeny was constructed with FastTree 2 [90]. In order to compute alpha and beta diversity, the ASVs table was rarefied at 16,000 reads to have an even number of reads across all the samples. Differences in alpha diversity (observations within sampling time points and dietary treatment) were evaluated by four indices: 1) Observed species index, which counts the numbers of ASV in each sample, also called richness; 2) Pielou’s evenness, which refers to the abundances of the species; 3) Shannon’s index which takes into account richness as well as how many of each ASV are observed (abundance), also called diversity; 4) Simpson’s index, which describes the diversity of a community. Two indices were used also for evaluation of beta diversity, which estimates the phylogenetic difference between bacteria communities: 1) Unweighted UniFrac Distance, indicating the number of different ASV and their phylogenetic distance; 2) Weighted UniFrac Distance, which takes into account the number of different ASV, their phylogenetic distance as well as the number of similar ASV.

### Statistical analysis and graphics

To evaluate the effect of the sampling time points through freshwater to seawater on gut microbiota composition and exclude the potential effect of functional ingredients, only fish fed Ref diets among sampling time points were analyzed and compared. At each sampling time point, statistical comparisons between Ref and Test diets were conducted to explore the effect of the functional ingredients. In order to assess differences in microbiota composition between the different treatments, the Kruskal-Wallis test followed by multiple comparisons was performed to compare the alpha diversity using GraphPad Prism 7 (GraphPad Software, La Jolla, California, United States). Regarding the dietary functional ingredients effect at SW2, the data of gut microbiota composition at phylum level was subjected to multiple t-tests using GraphPad Prism 7. In addition, Primer 7 (version, 7.0.13) was used to perform beta diversity analysis followed by PERMANOVA [91]. The raw data generated by QIIME2 was also used to make core microbiota of all samples at genus levels (above 0.1% relative abundance in 80% of samples) using MicrobiomeAnalyst [92]. The graphs of alpha diversity, heatmaps and gut microbiota composition were made by GraphPad Prism 7 basing on the raw data generated by QIIME2.

### Microbiome multivariable association with linear models (MaAsLin2)

Differentially abundant taxa (genus level) among the sampling time points and between the dietary treatments at SW2 were identified by the MaAsLin2 (version, 0.99.12) (https://huttenhower.sph.harvard.edu/maaslin2) in R, using the default program parameters. Bacterial taxa of very low abundance (< 0.01%) or low prevalence (present in < 25% of samples) were removed before running the differential abundance testing. The difference in the taxa abundance was considered significant when the *q*-value (FDR) was below 0.05.

Regarding the multivariate association analysis, the microbiota of distal intestinal digesta was tested for the associations with host responses (from the same individual fish) (Additional file 3: Table S2) using the MaAsLin2. Bacterial taxa of more than 0.1% abundance and 25% prevalence of samples were selected for association testing. The significant association was set at a *q*-value less than 0.25. The host responses, i.e. gut immune and barrier functions (gene expression in the distal intestine), as well as plasma nutrients (plasma cholesterol and triglyceride), were selected to run the multivariate association testing with fixed factor, i.e. treatment, since these gut immune and barrier functions, and plasma nutrients varied greatly among sampling time points with clearly decreasing values in fish at SW1 [40]. The gut immune functions related genes were selected for the association testing including the goblet cell marker (*muc13*), pro-inflammatory cytokines (interleukin-1 beta, *il1β* and interferon-gamma, *ifnγ*), anti-inflammatory cytokines (i.e. transforming growth factor-beta, *tgfβ* and interleukin 10, *il10*), T-cell markers (i.e. cluster of differentiation 3γδ and 8β, *cd3γδ* and *cd8β*), as well as the myeloid differentiation factor 88 (*myd88*). The gut barrier functions related genes were selected for the association testing including *zo-1*, *claudin-15* and *claudin-25b*. Since the expression levels of immune-related genes were highly correlated, we ran a principal component analysis (PCA) and extracted the first principal component (PC1) for the association testing to avoid multicollinearity and reduce the number of association testing. Similarly, gut barrier functions related genes were highly correlated, their extracted PC1 of the PCA was used for the association testing. The plasma nutrients were also highly correlated. Their extracted PC1 of the PCA was used for the association testing.

## Supplementary Information

**Additional file 1: Figure S1.** The bacterial DNA quantification among treatments. **Figure S2.** Microbial clades showing significant associations with expressions of barrier function related genes in the distal gut. Since the expression levels of barrier function related genes were highly correlated, we ran a principal component analysis (PCA) and used the first principal component (PC1) for the association testing to avoid multicollinearity and reduce the number of association testing. Except *Flavobacterium*, 26 differentially abundant taxa showed a clear negative correlation with expression levels of gut barrier function genes, which decreased as PC1 of the PCA increased. FDR, false discovery rate. **Figure S3.** The experimental conditions of salinity (A), temperature and oxygen (B) in water through the production cycle.

**Additional file 2: Table S1.** Relative abundance of all ASVs for each sample.

**Additional file 3: Table S2.** The metadata of interest for multivariate association analysis.

## Data Availability

Sequence data have been deposited at NCBI SRA database under BioProject accession ID: PRJNA660116.

## Abbreviations

LAB: Lactic acid bacteria
PERMANOVA: Permutation multivariate analysis of variance
PCoA: Principal coordinate analysis
16S rRNA: 16 Svedberg ribosomal ribonucleic acid
QIIME 2: Quantitative Insights Into Microbial Ecology 2
ASVs: Amplicon sequence variants
ANOVA: Analysis of molecular variance
il1β: Interleukin-1 beta
ifnγ: Interferon gamma
tgfβ: Transforming growth factor-beta
il10: Interleukin 10
cd3γδ: Cluster of differentiation 3γδ
cd8β: Cluster of differentiation 8β
myd88: Myeloid differentiation factor 88
PCA: Principal component analysis
PC1: Extracted the first principal component

## References

[1] Wang AR, Ran C, Ringø E, Zhou ZG (2018). Progress in fish gastrointestinal microbiota research. Rev Aquac.

[2] Tarnecki AM, Burgos FA, Ray CL, Arias CR (2017). Fish intestinal microbiome: diversity and symbiosis unravelled by metagenomics. J Appl Microbiol.

[3] Ray A, Ghosh K, Ringø EJAN (2012). Enzyme-producing bacteria isolated from fish gut: a review. Aquac Nutr.

[4] Falcinelli S, Picchietti S, Rodiles A, Cossignani L, Merrifield DL, Taddei AR (2015). *Lactobacillus rhamnosus* lowers zebrafish lipid content by changing gut microbiota and host transcription of genes involved in lipid metabolism. Sci Rep.

[5] Semova I, Carten JD, Stombaugh J, Mackey LC, Knight R, Farber SA (2012). Microbiota regulate intestinal absorption and metabolism of fatty acids in the Zebrafish. Cell Host Microbe.

[6] Ringø E, Zhou Z, Vecino JLG, Wadsworth S, Romero J, Krogdahl Å (2016). Effect of dietary components on the gut microbiota of aquatic animals. A never-ending story?. Aquac Nutr.

[7] Merrifield DL, Carnevali O. Chapter 8: Probiotic Modulation of the Gut Microbiota of Fish. In: Merrifield DL, Ringø E. editors. Aquaculture nutrition: gut health, probiotics and prebiotics. Wiley-Blackwell; 2014. p. 185-223.

[8] Austin B (2006). The bacterial microflora of fish, revised. Sci World J.

[9] Gajardo K, Jaramillo-Torres A, Kortner TM, Merrifield DL, Tinsley J, Bakke AM (2017). Alternative Protein Sources in the Diet Modulate Microbiota and Functionality in the Distal Intestine of Atlantic Salmon (*Salmo salar*). Appl Environ Microbiol.

[10] Zarkasi KZ, Taylor RS, Abell GC, Tamplin ML, Glencross BD, Bowman JP (2016). Atlantic Salmon (*Salmo salar* L.) gastrointestinal microbial community dynamics in relation to Digesta properties and diet. Microb Ecol.

[11] Catalan N, Villasante A, Wacyk J, Ramirez C, Romero J (2018). Fermented soybean meal increases lactic acid Bacteria in gut microbiota of Atlantic Salmon (*Salmo salar*). Probiotics Antimicrob.

[12] Jaramillo-Torres A, Rawling M, Rodiles A, Mikalsen HE, Johansen LH, Tinsley J (2019). Influence of dietary supplementation of probiotic *Pediococcus acidilactici MA18/5M* during the transition from freshwater to seawater on intestinal health and microbiota of Atlantic salmon (*Salmo salar* L.). Front Microbiol.

[13] Krogdahl Å, Kortner TM, Jaramillo-Torres A, Gamil AAA, Chikwati E, Li Y (2020). Removal of three proteinaceous antinutrients from soybean does not mitigate soybean-induced enteritis in Atlantic salmon (*Salmo salar*, L). Aquac..

[14] Dehler CE, Secombes CJ, Martin SA (2017). Environmental and physiological factors shape the gut microbiota of Atlantic salmon parr (*Salmo salar* L.). Aquac..

[15] Rud I, Kolarevic J, Holan AB, Berget I, Calabrese S, Terjesen BF (2017). Deep-sequencing of the bacterial microbiota in commercial-scale recirculating and semi-closed aquaculture systems for Atlantic salmon post-smolt production. Aquac Eng.

[16] Fogarty C, Burgess CM, Cotter PD, Cabrera-Rubio R, Whyte P, Smyth C (2019). Diversity and composition of the gut microbiota of Atlantic salmon (*Salmo salar*) farmed in Irish waters. J Appl Microbiol.

[17] Hovda MB, Fontanillas R, McGurk C, Obach A, Rosnes JT (2012). Seasonal variations in the intestinal microbiota of farmed Atlantic salmon (*Salmo salar* L.). Aquac Res.

[18] Zarkasi KZ, Abell GC, Taylor RS, Neuman C, Hatje E, Tamplin ML (2014). Pyrosequencing-based characterization of gastrointestinal bacteria of Atlantic salmon (*Salmo salar* L.) within a commercial mariculture system. J Appl Microbiol.

[19] Karlsen C, Ottem KF, Brevik OJ, Davey M, Sorum H, Winther-Larsen HC (2017). The environmental and host-associated bacterial microbiota of Arctic seawater-farmed Atlantic salmon with ulcerative disorders. J Fish Dis.

[20] Gajardo K, Rodiles A, Kortner TM, Krogdahl A, Bakke AM, Merrifield DL (2016). A high-resolution map of the gut microbiota in Atlantic salmon (*Salmo salar*): a basis for comparative gut microbial research. Sci Rep.

[21] Lokesh J, Kiron V, Sipkema D, Fernandes JMO, Moum T (2019). Succession of embryonic and the intestinal bacterial communities of Atlantic salmon (*Salmo salar*) reveals stage-specific microbial signatures. Microbiologyopen..

[22] Bjornsson BT, Stefansson SO, McCormick SD (2011). Environmental endocrinology of salmon smoltification. Gen Comp Endocrinol.

[23] Sundh H, Lindstrom J, Hasselberg-Frank L, Stefansson SO, McCormick SD, Nilsen TO (2014). Development of intestinal ion-transporting mechanisms during smoltification and seawater acclimation in Atlantic salmon *Salmo salar*. J Fish Biol.

[24] Handeland SO, Imsland AK, Bjornsson BT, Stefansson SO, Porter M (2013). Physiology during smoltification in Atlantic salmon: effect of melatonin implants. Fish Physiol Biochem.

[25] Tipsmark CK, Sorensen KJ, Hulgard K, Madsen SS (2010). Claudin-15 and -25b expression in the intestinal tract of Atlantic salmon in response to seawater acclimation, smoltification and hormone treatment. Comp Biochem Physiol A Mol Integr Physiol.

[26] Dehler CE, Secombes CJ, Martin SAM (2017). Seawater transfer alters the intestinal microbiota profiles of Atlantic salmon (*Salmo salar* L.). Sci Rep.

[27] Rudi K, Angell IL, Pope PB, Vik JO, Sandve SR, Snipen LG (2018). Stable Core Gut Microbiota across the Freshwater-to-Saltwater Transition for Farmed Atlantic Salmon. Appl Environ Microbiol.

[28] Lokesh J, Kiron V (2016). Transition from freshwater to seawater reshapes the skin-associated microbiota of Atlantic salmon. Sci Rep.

[29] Butt RL, Volkoff H (2019). Gut microbiota and energy homeostasis in fish. Front Endocrinol.

[30] Legrand TP, Wynne JW, Weyrich LS, Oxley AP (2020). A microbial sea of possibilities: current knowledge and prospects for an improved understanding of the fish microbiome. Rev Aquac.

[31] Egerton S, Culloty S, Whooley J, Stanton C, Ross RP (2018). The gut microbiota of marine fish. Front Microbiol.

[32] Tacchi L, Bickerdike R, Douglas A, Secombes CJ, Martin SA (2011). Transcriptomic responses to functional feeds in Atlantic salmon (*Salmo salar*). Fish Shellfish Immunol.

[33] Kiron V (2012). Fish immune system and its nutritional modulation for preventive health care. Anim Feed Sci Tech.

[34] Carbone D, Faggio C (2016). Importance of prebiotics in aquaculture as immunostimulants. Effects on immune system of *Sparus aurata* and *Dicentrarchus labrax*. Fish Shellfish Immunol..

[35] Torrecillas S, Montero D, Izquierdo M (2014). Improved health and growth of fish fed mannan oligosaccharides: potential mode of action. Fish Shellfish Immunol..

[36] Wang J, Lei P, Gamil AAA, Lagos L, Yue Y, Schirmer K (2019). Rainbow trout (*Oncorhynchus mykiss*) intestinal epithelial cells as a model for studying gut immune function and effects of functional feed ingredients. Front Immunol.

[37] Hossain MS, Koshio S, Kestemont P (2019). Recent advances of nucleotide nutrition research in aquaculture: a review. Rev Aquacu.

[38] Guerreiro I, Oliva-Teles A, Enes P (2018). Prebiotics as functional ingredients: focus on Mediterranean fish aquaculture. Rev Aquac.

[39] Ringø E, Olsen RE, Gifstad TO, Dalmo RA, Amlund H, Hemre GI (2010). Prebiotics in aquaculture: a review. Aquac Nut.

[40] Wang J, Kortner TM, Chikwati EM, Li Y, Jaramillo-Torres A, Jakobsen JV (2020). Gut immune functions and health in Atlantic salmon (*Salmo salar*) from late freshwater stage until one year in seawater and effects of functional ingredients: a case study from a commercial sized research site in the Arctic region. Fish Shellfish Immunol..

[41] Herlemann DPR, Labrenz M, Jurgens K, Bertilsson S, Waniek JJ, Andersson AF (2011). Transitions in bacterial communities along the 2000 km salinity gradient of the Baltic Sea. ISME J.

[42] Hoar W (1988). The physiology of Smolting Salmonids. Fish Physiol..

[43] Johansson LH, Timmerhaus G, Afanasyev S, Jorgensen SM, Krasnov A (2016). Smoltification and seawater transfer of Atlantic salmon (*Salmo salar* L.) is associated with systemic repression of the immune transcriptome. Fish Shellfish Immunol.

[44] Karlsen C, Ytteborg E, Timmerhaus G, Host V, Handeland S, Jorgensen SM (2018). Atlantic salmon skin barrier functions gradually enhance after seawater transfer. Sci Rep..

[45] Jin Y, Angell IL, Sandve SR, Snipen LG, Olsen Y, Rudi K (2019). Atlantic salmon raised with diets low in long-chain polyunsaturated n-3 fatty acids in freshwater have a *Mycoplasma*-dominated gut microbiota at sea. Aquac Env Interac.

[46] Ringø E, Gatesoupe FJ (1998). Lactic acid bacteria in fish: a review. Aquac..

[47] Ringø E, Lovmo L, Kristiansen M, Bakken Y, Salinas I, Myklebust R (2010). Lactic acid bacteria vs. pathogens in the gastrointestinal tract of fish: a review. Aquac Res.

[48] Balcázar JL, De Blas I, Ruiz-Zarzuela I, Vendrell D, Gironés O, Muzquiz JLJFI (2007). Enhancement of the immune response and protection induced by probiotic lactic acid bacteria against furunculosis in rainbow trout (*Oncorhynchus mykiss*). Fems Immunol Med Mic.

[49] Askarian F, Zhou Z, Olsen RE, Sperstad S, Ringø E (2012). Culturable autochthonous gut bacteria in Atlantic salmon (*Salmo salar* L.) fed diets with or without chitin. Characterization by 16S rRNA gene sequencing, ability to produce enzymes and in vitro growth inhibition of four fish pathogens. Aquac.

[50] Reveco FE, Øverland M, Romarheim OH, Mydland LTJA (2014). Intestinal bacterial community structure differs between healthy and inflamed intestines in Atlantic salmon (*Salmo salar* L.). Aquac.

[51] Schmidt V, Amaral-Zettler L, Davidson J, Summerfelt S, Good C (2016). Influence of fishmeal-free diets on microbial communities in Atlantic Salmon (*Salmo salar*) recirculation aquaculture systems. Appl Environ Microbiol.

[52] Webster TMU, Rodriguez-Barreto D, Castaldo G, Gough P, Consuegra S (2020). Garcia de Leaniz C. environmental plasticity and colonisation history in the Atlantic salmon microbiome: a translocation experiment. Mol Ecol.

[53] Heys C, Cheaib B, Busetti A, Kazlauskaite R, Maier L, Sloan WT (2020). Neutral Processes Dominate Microbial Community Assembly in Atlantic Salmon, *Salmo salar*. Appl Environ Microbiol.

[54] Bozzi D, Rasmussen JA, Carøe C, Sveier H, Nordøy K, Gilbert MTP (2020). Salmon Gut Microbiota Correlates With Disease Infection Status: Potential for Monitoring Health in Farmed Animals.

[55] Holben WE, Williams P, Gilbert M, Saarinen M, Sarkilahti LK, Apajalahti JH (2002). Phylogenetic analysis of intestinal microflora indicates a novel *Mycoplasma* phylotype in farmed and wild salmon. Microb Ecol.

[56] Llewellyn MS, McGinnity P, Dionne M, Letourneau J, Thonier F, Carvalho GR (2016). The biogeography of the Atlantic salmon (*Salmo salar*) gut microbiome. ISME J.

[57] Gupta S, Feckaninova A, Lokesh J, Koscova J, Sorensen M, Fernandes J (2019). *Lactobacillus* dominate in the intestine of Atlantic Salmon fed dietary probiotics. Front Microbiol.

[58] Gupta S, Lokesh J, Abdelhafiz Y, Siriyappagouder P, Pierre R, Sorensen M (2019). Macroalga-derived alginate oligosaccharide alters intestinal Bacteria of Atlantic Salmon. Front Microbiol.

[59] Webster TMU, Consuegra S, Hitchings M, Garcia de Leaniz C (2018). Interpopulation Variation in the Atlantic Salmon Microbiome Reflects Environmental and Genetic Diversity. Appl Environ Microbiol.

[60] Nguyen CDH, Amoroso G, Ventura T, Minich JJ, Elizur A. Atlantic Salmon (*Salmo salar* L., 1758) gut microbiota profile correlates with flesh pigmentation: cause or effect? Mar Biotechnol. 2020;22:786–804. 10.1007/s10126-019-09939-1 31942646

[61] Cheaib B, Yang P, Kazlauskaite R, Lindsay E, Heys C, De Noa M, et al. Unpicking the mysterious symbiosis of *Mycoplasma* in salmonids. bioRxiv. 2020.

[62] Song SK, Beck BR, Kim D, Park J, Kim J, Kim HD (2014). Prebiotics as immunostimulants in aquaculture: a review. Fish Shellfish Immunol..

[63] Mehdinejad N, Imanpour MR, Jafari V (2018). Combined or individual effects of dietary probiotic Pedicoccus acidilactici and nucleotide on growth performance, intestinal microbiota, Hemato-biochemical parameters, and innate immune response in goldfish (*Carassius auratus*). Probiotics Antimicrob Proteins.

[64] Dimitroglou A, Merrifield DL, Moate R, Davies SJ, Spring P, Sweetman J (2009). Dietary mannan oligosaccharide supplementation modulates intestinal microbial ecology and improves gut morphology of rainbow trout, *Oncorhynchus mykiss* (Walbaum). J Anim Sci.

[65] Ringø E, Erik Olsen R, Gonzalez Vecino JL, Wadsworth S (2012). Use of Immunostimulants and Nucleotides in Aquaculture: A Review. J Mar Sci Res Dev.

[66] Adel M, Lazado CC, Safari R, Yeganeh S, Zorriehzahra MJ (2017). Aqualase®, a yeast-based in-feed probiotic, modulates intestinal microbiota, immunity and growth of rainbow trout *Oncorhynchus mykiss*. Aquac Res.

[67] Rufchaie R, Hoseinifar SH (2014). Effects of dietary commercial yeast glucan on innate immune response, hematological parameters, intestinal microbiota and growth performance of white fish (*Rutilus frisii kutum*) fry. Croatian J Fisheries.

[68] Sakai MJA (1999). Current research status of fish immunostimulants. Aquac..

[69] Liu Z, Liu W, Ran C, Hu J, Zhou Z (2016). Abrupt suspension of probiotics administration may increase host pathogen susceptibility by inducing gut dysbiosis. Sci Rep.

[70] Tsilingiri K, Barbosa T, Penna G, Caprioli F, Sonzogni A, Viale G (2012). Probiotic and postbiotic activity in health and disease: comparison on a novel polarised ex-vivo organ culture model. Gut..

[71] Mileti E, Matteoli G, Iliev ID, Rescigno M (2009). Comparison of the immunomodulatory properties of three probiotic strains of *Lactobacilli* using complex culture systems: prediction for *in vivo* efficacy. PLoS One.

[72] Kononova SV, Zinchenko DV, Muranova TA, Belova NA, Miroshnikov AI (2019). Intestinal microbiota of salmonids and its changes upon introduction of soy proteins to fish feed. Aquac Int.

[73] Karlsson CL, Onnerfalt J, Xu J, Molin G, Ahrne S, Thorngren-Jerneck K (2012). The microbiota of the gut in preschool children with normal and excessive body weight. Obesity..

[74] Mazmanian SK, Round JL, Kasper DL (2008). A microbial symbiosis factor prevents intestinal inflammatory disease. Nature..

[75] Smith PM, Howitt MR, Panikov N, Michaud M, Gallini CA, Bohlooly YM (2013). The microbial metabolites, short-chain fatty acids, regulate colonic Treg cell homeostasis. Science..

[76] Osorio CR, Toranzo AE, Romalde JL, Barja JL (2000). Multiplex PCR assay for *ureC* and 16S rRNA genes clearly discriminates between both subspecies of *Photobacterium damselae*. Dis Aquat Org.

[77] Zhao DH, Sun JJ, Liu L, Zhao HH, Wang HF, Liang LQ (2009). Characterization of two phenotypes of *Photobacterium damselae* subsp. *damselae* isolated from diseased juvenile *Trachinotus ovatus* reared in cage mariculture. J World Aquac Soc.

[78] Maynard CL, Elson CO, Hatton RD, Weaver CT (2012). Reciprocal interactions of the intestinal microbiota and immune system. Nature..

[79] Bakke-McKellep AM, Nordrum S, Krogdahl Å, Buddington R (2000). Absorption of glucose, amino acids, and dipeptides by the intestines of Atlantic salmon (*Salmo salar* L.). Fish Physiol Biochem.

[80] Knudsen BE, Bergmark L, Munk P, Lukjancenko O, Prieme A, Aarestrup FM, et al. Impact of Sample Type and DNA Isolation Procedure on Genomic Inference of Microbiome Composition. mSystems. 2016;1(5):e00095-16. 10.1128/mSystems.00095-16 PMC5080404 27822556

[81] Vandeputte D, Kathagen G, D’hoe K, Vieira-Silva S, Valles-Colomer M, Sabino J (2017). Quantitative microbiome profiling links gut community variation to microbial load. Nature..

[82] Ramseier CA, Kinney JS, Herr AE, Braun T, Sugai JV, Shelburne CA (2009). Identification of pathogen and host-response markers correlated with periodontal disease. J Periodontol.

[83] Bolyen E, Rideout JR, Dillon MR, Bokulich N, Abnet CC, Al-Ghalith GA (2019). Reproducible, interactive, scalable and extensible microbiome data science using QIIME 2. Nat Biotechnol.

[84] Callahan BJ, McMurdie PJ, Rosen MJ, Han AW, Johnson AJA, Holmes SP (2016). DADA2: high-resolution sample inference from Illumina amplicon data. Nat Methods.

[85] Quast C, Pruesse E, Yilmaz P, Gerken J, Schweer T, Yarza P (2013). The SILVA ribosomal RNA gene database project: improved data processing and web-based tools. Nucleic Acids Res.

[86] Bokulich NA, Kaehler BD, Rideout JR, Dillon M, Bolyen E, Knight R (2018). Optimizing taxonomic classification of marker-gene amplicon sequences with QIIME 2’s q2-feature-classifier plugin. Microbiome..

[87] Davis NM, Proctor DM, Holmes SP, Relman DA, Callahan BJ (2018). Simple statistical identification and removal of contaminant sequences in marker-gene and metagenomics data. Microbiome..

[88] Parris DJ, Brooker RM, Morgan MA, Dixson DL, Stewart FJ (2016). Whole gut microbiome composition of damselfish and cardinalfish before and after reef settlement. Peerj..

[89] Katoh K, Misawa K, Kuma K, Miyata T (2002). MAFFT: a novel method for rapid multiple sequence alignment based on fast Fourier transform. Nucleic Acids Res.

[90] Price MN, Dehal PS, Arkin APJ (2010). FastTree 2–approximately maximum-likelihood trees for large alignments. PLoS One.

[91] Clarke KR, Clarke K, Gorley K, Clarke K, Gorley R (2006). PRIMER v6: user manual/tutorial.

[92] Dhariwal A, Chong J, Habib S, King IL, Agellon LB, Xia J (2017). MicrobiomeAnalyst: a web-based tool for comprehensive statistical, visual and meta-analysis of microbiome data. Nucleic Acids Res.

