# Supplementary material for: Microbiota in intestinal digesta of Atlantic salmon (Salmo salar), observed from late freshwater stage until one year in seawater, and effects of functional ingredients: a case study from a commercial sized research site in the Arctic region
Source: Anim Microbiome. 2021 Jan 28;3:14. doi: 10.1186/s42523-021-00075-7 (PMC7841887; doi:10.1186/s42523-021-00075-7)
Supplement: Supplementary file 1 — Additional file 1: Figure S1. The bacterial DNA quantification among treatments. Figure S2. Microbial clades showing significant associations with expressions of barrier function related genes in the distal gut. Since the expression levels of barrier function related genes were highly correlated, we ran a principal component analysis (PCA) and used the first principal component (PC1) for the association testing to avoid multicollinearity and reduce the number of association testing. Except Flavobacterium, 26 differentially abundant taxa showed a clear negative correlation with expression levels of gut barrier function genes, which decreased as PC1 of the PCA increased. FDR, false discovery rate. Figure S3. The experimental conditions of salinity (A), temperature and oxygen (B) in water through the production cycle. [file 42523_2021_75_MOESM1_ESM.docx]

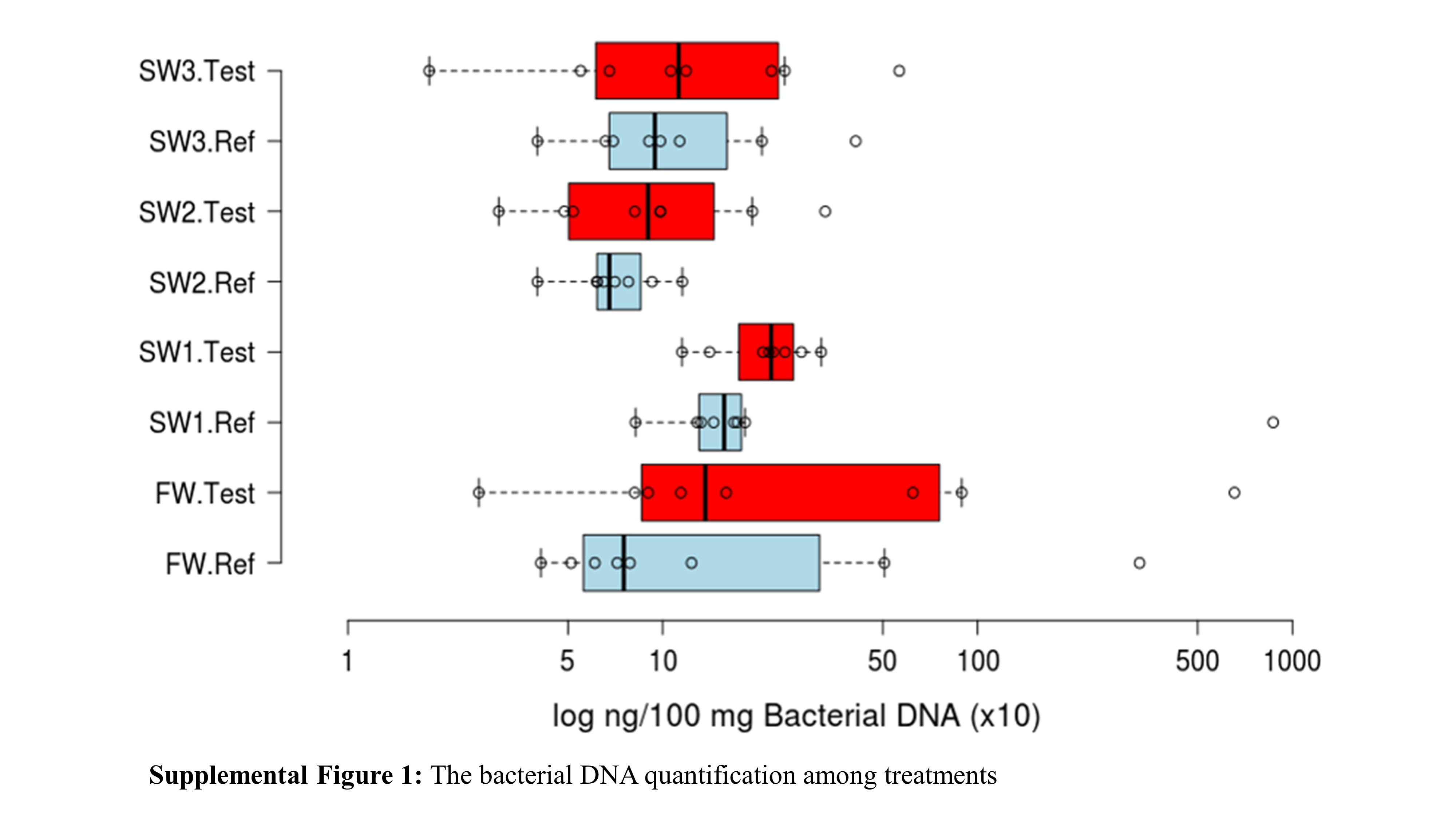


**Figure S1.** The bacterial DNA quantification among treatments.


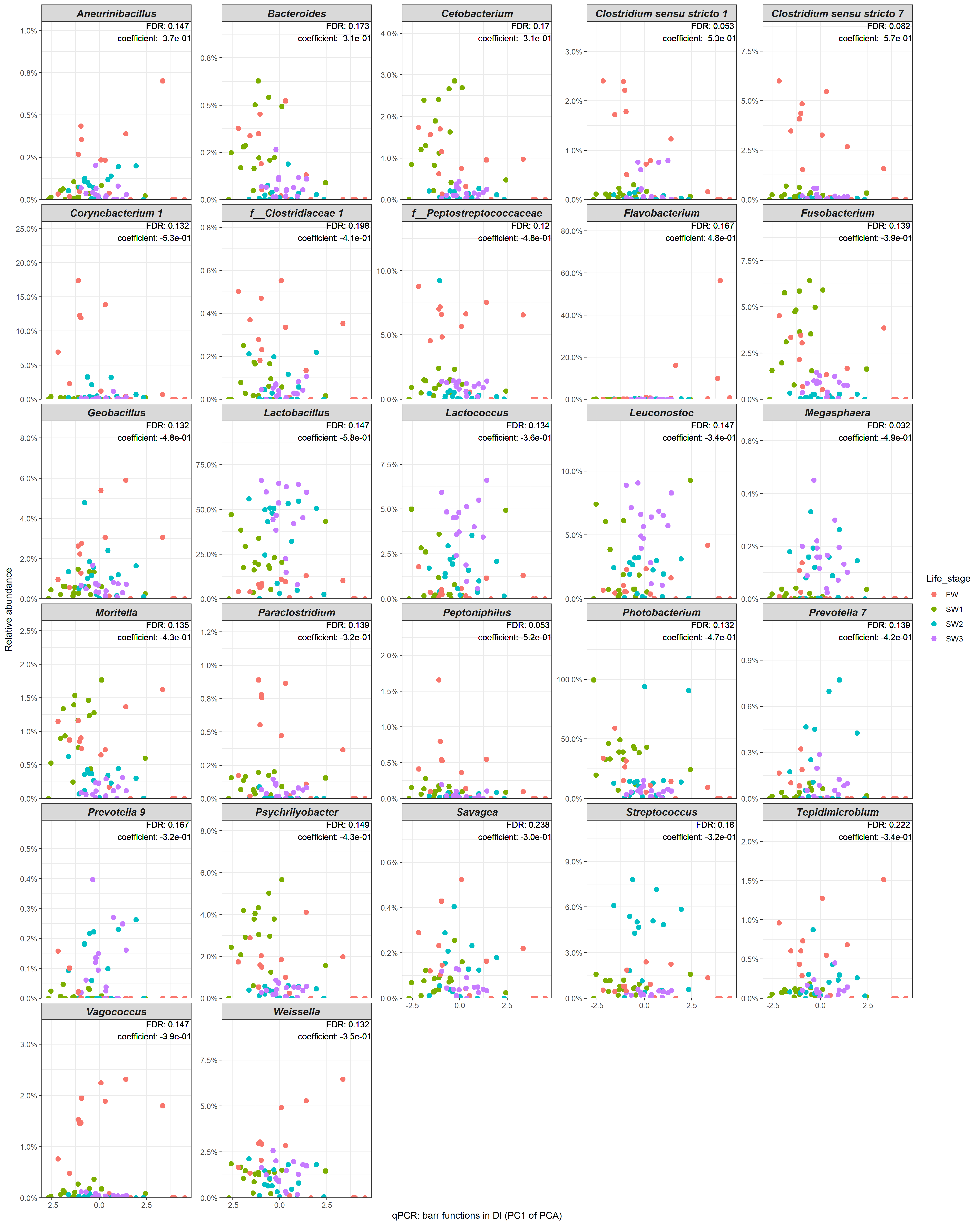


**Figure S2.** Microbial clades showing significant associations with expressions of barrier function related genes in the distal gut. Since the expression levels of barrier function related genes were highly correlated, we ran a principle component analysis (PCA) and used the first principle component (PC1) for the association testing to avoid multicollinearity and reduce the number of association testing. Except *Flavobacterium*, 26 differentially abundant taxa showed a clear negative correlation with expression levels of gut barrier function genes, which decreased as PC1 of the PCA increased. FDR, false discovery rate.


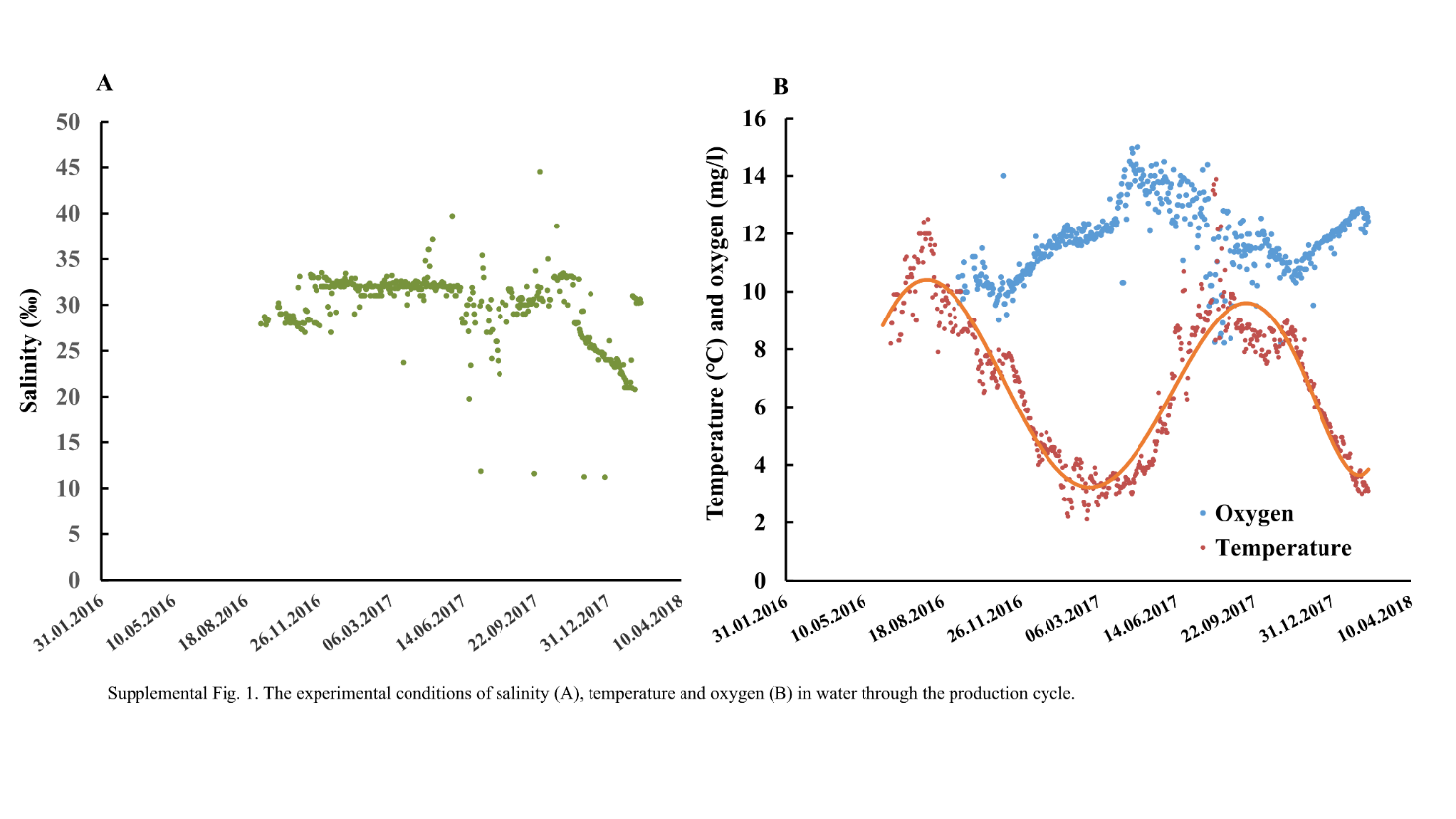


**Figure S3.** The experimental conditions of salinity (A), temperature and oxygen (B) in water through the production cycle.
